# Sustainable Isolation of Bioactive Compounds and Proteins from Plant-Based Food (and Byproducts)

**DOI:** 10.3390/plants12162904

**Published:** 2023-08-09

**Authors:** Zakir Showkat Khan, Saira Amir, Tea Sokač Cvetnić, Ana Jurinjak Tušek, Maja Benković, Tamara Jurina, Davor Valinger, Jasenka Gajdoš Kljusurić

**Affiliations:** 1 Department of Food Science and Technology, Guru Nanak Dev University, Amritsar 143005, India; 2 Department of Food Technology, School of Applied and Life Sciences, Uttaranchal University, Dehradun 248007, India; 3 Department of Nutrition Sciences, School of Health Sciences, University of Management and Technology, C-II Johar Town, Lahore 54700, Pakistan; 4 Faculty of Food Technology and Biotechnology, University of Zagreb, Pierottijeva ul. 6, HR-10000 Zagreb, Croatia

**Keywords:** plant-based food, byproducts, sustainable extraction, proteins, bioactive compounds

## Abstract

Plant-based food produces significantly less greenhouse gases, and due to its wealth of bioactive components and/or plant-based protein, it becomes an alternative in a sustainable food system. However, the processing and production of products from plant sources creates byproducts, which can be waste or a source of useful substances that can be reused. The waste produced during the production and processing of food is essentially nutrient- and energy-rich, and it is recognized as an excellent source of secondary raw materials that could be repurposed in the process of manufacturing and preparing food, or as feed for livestock. This review offers an overview of the sources and techniques of the sustainable isolation of bioactive substances and proteins from various sources that might represent waste in the preparation or production of food of plant origin. The aim is to uncover novel approaches to use waste and byproducts from the process of making food to provide this waste food an additional benefit, not forgetting the expectations of the end user, the consumer. For the successful isolation of bioactive ingredients and proteins from food of plant origin, it is crucial to develop more eco-friendly and efficient extraction techniques with a low CO_2_ footprint while considering the economic aspects.

## 1. Introduction

The production of human food has an extremely large impact on the environment, which can be measured through the emission of greenhouse gases (GHG) [1], and expressed in terms, it is about 13.6 billion tons of CO_2_ equivalent (or 26% of the total CO_2_ equivalent). Land use produces 1.09 billion tons of CO_2_ equivalent (8%), food crops (excluding food animals) account for 2.86 billion tons (21%), and the share of the food supply chain is 2.45 billion tons or 18% CO_2_ equivalent (which includes transport (6%); packaging (5%); food processing (4%); and retail (3%)) [2]. Analysis of the developed farm-to-fork strategy has shown an extremely positive effect on the reduction of primary food production impact on the environment with the aim of adapting to climate change. The strategy is aimed at economics, ecological well-being, and ethical awareness (of producers and consumers). The goal of the strategy is to achieve the aforementioned benefits by (i) reducing the impact of primary production on the environment and climate, while ensuring an economic return for growers, fishermen, and aquaculture producers, but also (ii) improving animal welfare and protecting plant health, and (iii) promoting the adoption of new green business models, i.e., a circular economy that is primarily focused on sustainability [3]. As defined in the strategy, “All stakeholders in the food chain must play their role in achieving its sustainability” [3], and the scientific community, which in any segment is related to food, is invited to create new solutions (greener analytical methods and production processes, reuse of food processing waste, etc.) that will contribute to sustainability.

The waste generated during food production and processing is basically rich in nutrients and a good source of energy. Furthermore, it is recognized as a source of secondary raw materials that can be reused in the process of food production and processing or as animal feed. Energy and nutritionally rich waste is generated in the processing of fruits/vegetables and oilseeds (such as grape pomace, cakes, etc.), which can be used as raw materials [4] to obtain some other products (e.g., proteins, pectin, ethanol, flavonoids, polyphenols, etc.). Namely, in this segment, the cooperation of the scientific community and entities in food production is the key in order to find new ways of using waste and byproducts from the food production process to give this waste food added value [5] in accordance with new Green Deal solutions for sustainable food processing [6].

Consumers are increasingly becoming responsible segments in preserving the environment and are turning to food and products that have a smaller CO_2_ footprint [7], which includes a greater representation of foods of plant origin from regional cultivation, but also those produced with the use of reused components, or upcycled foods, that are isolated from waste [8] from the production process of foodstuffs [9] and are of plant origin [10]. 

Therefore, the aim of this work is to provide an overview of the sources and methods of isolation of bioactive components and proteins from plant-based foods and those sources that potentially represent waste (byproducts) in the processing or production of food of plant origin.

## 2. Processing of Waste Plant-Food Production

Plant-based food industrial byproducts, often characterized as waste and used as animal feed [11], include the rest of foods such as: leaf, seed, shell, peel, stem, bran, kernel, pomace, oil cake, etc. [12]. The potential to reuse this waste is based on its richness in bioactive compounds and proteins [13].

**Figure 1:**
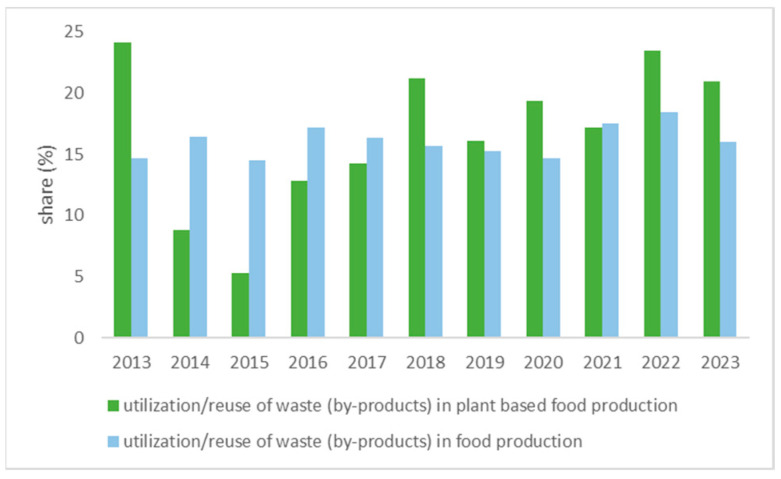
Share of publications that studied the use of waste/byproducts in (i) topics dealing with food production in general (■) and (ii) food production of plant origin, according to Web of Science (WoS, core collection) (■).

A comprehensive systematic review of the important scientific articles published on the topic of utilization/reuse food production waste/byproducts in the food production vs plant-based food production was conducted by using the core collection in the WoS (Web of Science database), including the last 10 years (2013–2022), adding also the information from the first six months of 2023. Key words used were “food production” or “plant-based food production” with refined results by the use of words such as “waste” or “by-products”, refined with “reuse” or “utilization”. Results are presented in Figure 1.

As presented in Figure 1, the share of reuse of byproducts and/or waste is in favor of plant-based food production, whose share is significantly higher than the same topics related to food production reuse of byproducts in general (*p* = 2 × 10^−4^). Food and drink labeled as “vegetable” is the focus of almost half of consumers (48%) [14], and 25% of all consumers declared that they increased their intake of plant-based proteins compared to the previous year (research conducted in 2021 by International Food Information Council [15]. The Western diet is low in fruits and vegetables (which results in low intake of vitamins, minerals, and diversity of amino acid intake) [16,17,18] and rich in refined carbohydrates, sugars, fats, and processed foods, as well as animal-sourced foods [19,20]. Therefore, it is not surprising that consumers are increasingly interested in food of plant origin, but also in food supplements that are sustainable and come from byproducts of plant-based food production [16,17]. An overview of the most represented byproducts (waste) in the processing of food of plant origin is given in Table 1, and includes byproducts of fruit processing, oil production, mill-bakery, and sugar industries.

The sources of food waste production are significantly bigger than what is mentioned in Table 1. However, the above represents the sources of exceptional amounts of production waste, so the world’s largest fruit crop is grapes (Vitis sp., Vitaceae), mostly (about 80% of the total crop) used in wine production [42]. However, what needs to be highlighted here is the fact that those concentrations of bioactive compounds (Table 1) represent some average values that may differ due to a change in detection method or as follows for the example of bioactive components in the byproduct of grape processing in winemaking, which makes up almost 20% of the initial mass in winemaking [52,53]. Scientific and professional research supports the pharmacological application and therapeutic benefits of a number of bioactive components, especially those with byproducts containing proanthocyanidins, e.g., grape seeds [53], which have been proven to contain a high proportion of antioxidants. Antioxidants are proven to be effective in minimizing oxidative stress, inflammatory processes, pathology associated with metabolic syndrome, and immediately after that with obesity [53]. The study, which indicates exceptional ranges of expected content of bioactive components in the byproduct, investigated 11 different remains of wine production in Argentina, which represents almost 5% of the total world wine production [54]. Fontana and coworkers [43] showed the variation of investigated bioactive compounds where, for example, the mentioned Quercetin (in Table 1) can, in freeze-dried grape pomace extracts, range from 218 μg per g (Cabernet Franc 2016, from the northern location of Altamira) to 1695 (Cabernet Sauvignon 2016, location of Agrelo). In two examined extracts, Quercetin was not detected (Cabernet Sauvignon 2016 and Cabernet Franc 2016, both from the Altamira location) [55]. In addition, this study presented also a large number of non-anthocyanin components, such as: (i) Hydroxybenzoic acids (Gallic acid, Syringic acid); (ii) Hydroxycinnamic acids (Caffeic acid, p-Coumaric acid, Ferulic acid); (iii) Stilbenes (trans-Resveratrol); (iv) Flavanols (Procyanidin B1, (+)-Catechin, Procyanidin B2, (-)-Epicatechin, (-)-Gallocatechin, (-)-Gallocatechin gallate, (-)-Epicatechin gallate); (v) Flavonols (Kaempferol-3-glucoside, Quercetin); and (vi) Other compounds (OH-tyrosol, Tyrosol). The important outcome is the range of total non-anthocyanins ranging from 2.813 mg/g to six times more, 16.853 mg/g of grape pomace extracts, which were determined in the same grape variety (Cabernet Sauvignon, harvested 2015 from Gualtallary vs. 2016 from the Agrelo location, respectively). The importance of the location is confirmed in Croatian vineyards, where the composition of grapes and pomace is different for the autochthonous variety Maraština, and it is a consequence of the dominant autochthonous microbiota in different vineyards (grapes from 11 vineyards were analyzed) [56].

Grains are considered to be under plant-based food, as well [57], therefore Table 2 is given as an overview of concentrations of bioactive compounds of different grain byproducts.

Table 1 states that phlorizin, which is present in numerous plants (present also in apple pomace), has been used in human medicine since ancient times [65]. Considering the obesity pandemic and the increased incidence of diabetes [66], it is precisely the antidiabetic effect of phlorizin that has shown improved hyperglycemia, and studies [67,68] have reported its anti-inflammatory and antioxidant effects [69]. Taking into account the exceptional contribution of bioactive compounds from plants and their medicinal properties, as well as consumer expectations to eat what will be good for health [70], all the listed bioactive components in Table 1 and Table 2 have their own benefits, arising from their antioxidant and anti-inflammatory properties. Cereals and grains also have their health effects based on bioactive compounds, such as antioxidant and antiproliferative activities [71] and functional ingredients [72]. However, the additional focus is on the sustainability of the environment and the production of cosmetics and food, and science is turning to the additional study of alternative analyses and extractions.

Therefore, for the extraction of bioactive compounds and proteins from plant-based food production waste, it is certainly necessary to weigh the pros and cons, keeping primarily profitability in mind [71,72].

Due to the obvious environmental benefits of limiting the use of food sourced from animals, plant-based diets are growing in popularity, especially in advanced nations [73]. There are numerous environmental costs associated with the processing and consumption of animals, including greenhouse gas emissions (GHGEs), land use and degradation, water consumption, nutrient contamination (such as an increase in acidity and eutrophication), the use of fertilizers and chemicals, and consumer-level food waste at every stage of the supply chain [74,75]. Among them, GHGEs, land destruction, and water use have significant upstream impacts on the environment. They amplify the effect of climate change and ultimately determine subsequent environmental degradation ((such as the likelihood of catastrophes, loss of biodiversity and habitat, shortage of clean water, etc.) in ecological systems [76]. 

### 2.1. Sustainable Isolation of Bioactive Compounds from Plant-Based Food Byproducts

In recent years, there has been an interest in “green” extraction techniques, which are primarily characterized by the minimization of volatile organic solvents and toxic substances and are united under the name green analytics or green analytical chemistry [77,78]. Conventional methods of extraction (e.g., Soxhlet extraction, digestion, maceration, etc.) are some of the techniques used to isolate bioactive compounds not only from food waste, but also from production byproducts, which represent high added value. However, the mentioned methods of extraction, due to their low efficiency, consumption of energy, time, and organic solvents, have a negative effect on the environment [79]. These are the reasons why they are not considered economical, and thus do not belong among sustainable techniques. On the other hand, the just-mentioned limitations influenced the development of new extraction procedures that are sustainable since they use green solvents and at the same time enable more efficient extractions with minimal proportions of ballast substances [80]. 

Thus, ref. [81] highlighted extraction (i) assisted by ultrasound, (ii) assisted by microwaves, (iii) supercritical fluid, (iv) liquid under pressure, and (v) assisted by enzymes and combinations as green methods of extraction of bioactive components. The advantage of the mentioned techniques is in the minimization or elimination of the use of toxic organic solvents while increasing the quality of the extract, as well as the utilization of the extraction [82]. With these techniques, the temperatures are much lower (they are often called “cold” extractions), and this consequently stabilizes the extracted bioactive compounds and certainly contributes to the high added value of food waste or byproducts that are reused [83].

### 2.2. Sustainable Isolation of Plant Proteins

Proteins play a crucial structural and nutritional role in food. They not only supply a variety of amino acids required for human health, but they also operate as thickening, stabilizing, emulsifying, foaming, gelling, and binding agents. The potential of a protein to possess and display such unique functional qualities is largely determined by its inherent structure, configuration, and the manner in which it interacts with other dietary ingredients such as polysaccharides, lipids, and polyphenolic compounds. Animal proteins are more functional, more easily digestible, and contain fewer antinutrients than plant proteins [84].

However, consumer preferences are changing towards clean, cruelty-free, vegan, or vegetarian plant-based foods that are obtained ethically and responsibly. In contrast to proteins derived from animals, plant proteins are more adaptable, accepted by consumers who are vegetarians and vegan on a religious and cultural level, and they require less water, soil, and waste during food processing [85]. Consequently, processing and using plant proteins has attracted global attention. A large number of scientific studies are concentrating on improving the use of plant proteins in food and pharmaceutical products through a variety of processing and modification techniques to enhance their functional properties, bioactivity, bioavailability, and digestibility [86]. From an agricultural and environmental sustainability perspective, plant-based products have a significant advantage. Employing plant-derived materials preserves the wellbeing of animals and also has an enhanced holistic social reputation. As a result, consumer demand and environmental concerns are driving a shift away from animal-based products and towards those made of plants. While some investigations have been done to characterize novel plant-based proteins, more work has to be carried out to determine effective protein extraction techniques that will result in high outputs while maintaining the functionality and quality of the extracted proteins (Table 3).

#### 2.2.1. Source of Plant-Based Proteins—Cereals 

On average, maize contains 72% carbohydrates, 10% proteins, and 4% fats [104]. The two primary products made from maize are meal and flour. A total of 70% of the world’s ethanol output comes from the wet and dry milling of maize [105]. Different byproducts, such as corn syrup, germ meal, corn oil, and corn gluten meal, are produced during the wet-milling process, whereas dried grains are produced during the dry-milling process [106]. Due to their high fiber and protein content, dried grains are currently used as animal feed [107,108]. 

While corn gluten feed provides up to 25% protein, corn gluten meal generated by wet milling has around 70% protein content. Protein content in corn syrup from dry-grinding plants can reach 40% of the dry mass [108]. Wheat bran contains from 13% to 18% protein and is startlingly high in arginine and lysine content, and it is considered as a reliable and good source for protein extraction. Additionally, the bran contains significant amounts of cysteine, tyrosine, and tryptophan [109]. 

#### 2.2.2. Source of Plant-Based Proteins—Oilseeds

Seeds continue to be the principal source of vegetable oil used by both humans and animals, in addition to providing crucial components for modern, commercial, and combustion uses. This has led to an ever-increasing demand for vegetable oils on a global basis [110]. 

About 364 million tons of byproducts, which include meals or cakes with high-value components including cellulose, polysaccharides, proteins, phenolic compounds, etc., are produced during the processing of vegetable oils [110]. Remaining cakes have about 50% protein depending on the kind of plant residues used [111]. After processing rapeseed for oil, the resulting cake is highly rich in proteins (28–31% in the case of cold pressing and 38–45% in the case of hot pressing). Similarly, sunflower meal, produced after oil extraction, contains 20–30% of protein and various techniques can be applied to extract these proteins [112].

#### 2.2.3. Source of Plant-Based Proteins—Microalgae

In societies with a shortage of animal-based proteins or where maintaining an appropriate ratio of essential amino acids is difficult, proteins from microalgae can be utilized to restore a person’s protein intake. The enhancement of human wellness may stem from microalgal proteins, especially in vegans and vegetarians [113]. Using microalgal proteins will also relieve the pressure on resource-intensive land crops for food [114].

Humans have been using them as nourishment for many years. There are thought to be over 200,000 different species of microalgae, making them a remarkably diverse collection of microbes [115]. The *Cyanophyceae*, *Bacillari-ophyceae*, *Chrysophyceae*, and *Chlorophyceae* are among the most well-known and potentially more ecologically significant [116]. Differed microalgal species might have quite varied protein concentrations and are significantly impacted by the surroundings where they are cultivated. High quantities of protein, generally 40–60% of dry matter, can occur in numerous species [117]. Based on dry mass, the variety of crude protein concentrations in the biomass of microalgae is 6–63%, with over 40% in many species [118]. The protein content of 17 different microalgal species ranging from 6 to 58% has also been reported [119]. Hence, microalgae can act as a sustainable source of protein if used correctly.

#### 2.2.4. Source of Plant-Based Proteins—Millet Proteins

Millets are tiny seeds that grow in semi-arid and dry land regions around the world. They may thrive in dry, hot, and humid areas where growing wheat and rice is highly un-likely [120]. Contrary to traditional basic cereals, these are more reasonably priced and contain a higher amount of nutrients, such as carbohydrates, protein, dietary fiber, and minerals like calcium, iron, magnesium, and phosphorus, as well as a number of trace minerals [121]. Proteins make up roughly 10% of the weight of millet [120]. Compared to wheat and rice, the overall protein level of different millet grain varieties varies from 7.52% to 12.1% [122]. Leucine, phenylalanine, proline, serine, tyrosine, aspartic acid, and glutamic acid are among the amino acids that are present in greater concentrations in millet protein [123]. It has great potential for producing low-cost protein extracts that may be used in the food business. 

Plant-based foods are becoming more popular as they serve an important part in sustainable, low-meat, and nutritious diets. A growing segment of the population is converting to a plant-based diet due to the positive effects on their health and the environment. Research has shown that eating a diet high in fruits, vegetables, legumes, nuts, and whole grains may help prevent lifestyle-related illnesses like heart disorders, type II diabetes, and breast, colon, and other digestive-related cancers, as well as enhance psychological well-being. Long-term research examining the spectrum of plant-based foods and their effects on human well-being is required. The findings would make it possible to formulate dietary recommendations for plant-based meals, particularly for those at risk, including small children, pregnant women, and elderly people, and are more likely to experience nutritional deficiencies.

## 3. Challenges of Plant Proteins Use

Animal proteins are regarded as high-quality or complete proteins because they have all of the essential amino acids, but plant proteins are seen as incomplete since some of the important amino acids required by humans for healthy growth are missing. Certain remarkable plant proteins, such as soybean and the pseudo cereals quinoa and amaranth, contain all nine essential amino acids [124]. Plant-based proteins with low protein content and absence of critical amino acids are only scarcely used in food items, but several modification techniques, including chemical, physical, and enzymatic procedures, improve their usability. 

Plant proteins can be altered using a variety of methods, which alter their physicochemical and functional characteristics as well as fix some of their drawbacks. This opens the door for the development of plant proteins as multifunctional food system elements. Furthermore, mixed protein systems have been researched to improve protein characteristics, primarily employing legumes and dairy proteins [124]. Further issues for the food business in producing final products include high-quality protein with good flavor, texture, color, and mouth feel, as well as reasonable cost of plant protein. Byproducts from many different crops have recently been used to extract the protein in order to solve the availability issue; one example is the protein extraction from sesame bran waste using a microwave and enzymes [125] and protein extraction from tea leftovers and other dietary waste or byproducts [126]. Another obstacle to the use of plant proteins is their potential toxicity, such as the toxicity of gossypol in cottonseed protein [127]. 

## 4. Protein Isolation

Protein isolation is a series of steps, with each step increasing the purity of the isolate. These steps may include identification of the source, extraction, separation from non-protein components, and concentration. 

The type of source influences the different extraction methods used. By using cutting-edge protein extraction procedures, protein isolates abundant in crucial amino acids, increased physicochemical, as well as functional qualities can be produced. 

The conventional techniques for protein isolation can be used with a larger range of plants, such as oilseeds, agro-residues, cereals, and pulses, but they have significant drawbacks (expensive, unsustainable) [128]. It has been discovered that pulsed electric field extraction is a method for isolating recombinant enzymes from microorganisms in their natural conformation while maintaining a high level of the specific activity. 

Due to their numerous advantages (better yield, quicker, more environmentally friendly, and using less solvent) over traditional procedures, modern protein extraction techniques are the method of choice. Due to improved extraction recoveries, it is becoming increasingly common nowadays to successively utilize these innovative methods for the isolation of proteins. These techniques are broadly divided into two categories, conventional and novel green extraction procedures. Conventional extraction is further classified into dry extraction and wet extraction [129,130,131].

### 4.1. Dry Extraction

Air classification is one of the most commonly used dry extraction techniques. Conducted studies demonstrated that air classification may group cereal and pulse flours into subsets with various nutrient content and sizes of particles. Using this method, a classifier chamber receives air currents. The feed flour is separated into tiny and coarse bits with distinct dimensions and densities within the chamber by the gravitational and centrifugal forces of air. In order to increase the effectiveness of separation, reduction of the size of the feed material usually occurs before air classification [132]. 

Hammer milling, attrition milling, pin milling, and jet milling are a few examples of milling and size reduction processes. In more recent studies, electrostatic fractionation of solids has been examined in addition to air classification. Instead of variances in size and density, this approach depends on variations in the dielectric characteristics of the feed particles [133]. It is discovered that electrostatic separation can increase protein extraction by up to 15% compared to air classification [134].

### 4.2. Wet Extraction 

#### 4.2.1. Enzyme-Based Extraction

The growing demand for premium protein-rich products has prompted the development of non-traditional sources of proteins, such as algae, plant waste, fungi, and food industry waste, that are not harmful to the environment [135]. Protein is recovered from grain and oilseed residues. Following the dehulling process, or defatting, these are reported to contain 15–20% protein content [136]. Substituting plant proteins derived from food manufacturing wastes for animal proteins has ensured food production sustainability, and the type of protein extraction used is dictated by the final product’s intended implementation. Enzymatic protein extraction is suitable for use in emulsion-based products [137]. 

Enzyme-assisted extraction (EAE) is a viable method for extracting high-quality protein from plants sustainably. The recovery of protein from cells is made difficult by a stiff cell wall. EAE mainly deals with undermining cell wall stability through the enzymatic breakdown of cell wall components. Cell wall dissolution performed by pectinases and carbohydrates contributes to the regulated extrusion of protein from cells in oilseed, legumes, and cereal seeds [138]. Proteases increase protein output by releasing proteins from the polysaccharide structure. Cell wall breakdown promotes cellular protein outflow. Following protein release, proteases fractionate higher molecular mass proteins into smaller but more soluble segments, resulting in favorable extraction parameters. Furthermore, proteases function at optimal pH levels, minimizing the denaturation of proteins. 

A common protease concentration of 1–5% g/g substrate is regarded as optimum for different extraction methods. Under certain circumstances, such enzymes may additionally hinder the buildup of complexes between discharged proteins and different parts of cells, such as carbohydrates and phytates [139]. Along with EAE, physical treatments such as ultrasound, sonication, and microwave boost both the quantity and quality of the extract [104]. Press cakes prepared using dehulled rapeseeds are treated with pectinolytic, cellulolytic, and xylanolytic enzyme mixtures to extract proteins. Proteolytic enzymes dissolved the cellular barriers through hydrolysis of pectic and glucans polysaccharides, significantly boosting the overall protein extract rate by 1.7-fold. A 74% protein extraction yield was obtained from intact seeds and 56% from dehulled rapeseed press cakes [138]. Defatted barley flour was enzymatically treated with (1) amyloglucosidase and α-amylase, and (2) amyloglucosidase, β-1,3,4-glucanase, and α-amylase. The first method produced a protein concentrate of 49.0%, while the second method produced 78.3% protein prior to the precipitation process [140,141]. 

The product outputs may be improved even more if this approach is implemented in a sequence using mechanical protein extraction methods. Despite the fact that EAE has multiple limitations, such as time, being functionally expensive, being tricky in expanding up the extraction process, having incoherent yields, and being a costly technique to operate, it is still regarded as effective because it has a low environmental impact while producing a substantial protein output. This extraction technology produces high-quality food items appropriate for human consumption. Furthermore, reusing (immobilized catalysts) could significantly lower the cost of protein extraction with this technology. 

#### 4.2.2. Aqueous Two-Phase System

Aqueous two-phase systems (ATPS) are now employed for effective protein isolation due to the qualities they have, including phase system hydrophobicity, between phases electrical potential, size of molecules, and protein bio-affinity [142]. Protein separation, concentration, and purification are all possible using the multipurpose ATPS approach that relies on the blending of two elements with differing compositions, where a proper selection of these two elements ensures the end result of two specified and balanced layers. 

Scientific researchers are very interested in the advantages of ATPS technology, which, in comparison with traditional mechanisms, ensures a higher level of purity, selectivity, and extraction yield thanks to its flexibility, biocompatibility, and economic ease. Furthermore, phase components can stabilize protein frameworks while maintaining the biological function of the proteins [142]. Recent studies have shown that two-phase systems made of polyethylene glycol and sodium citrate or ethanol and sodium citrate are effective at recovering proteins from waste microalgae and prawns [142,143,144,145].

#### 4.2.3. Reverse Micelles Extraction (RM)

Traditional protein extraction techniques have a number of limitations, including rapid acceleration of protein denaturation, production of enormous volumes of wastewater, and restricted ability to handle raw materials. The technology used by RM to extract dietary proteins is becoming more and more popular. When extracting proteins using the RM method, there are often two processes involved: a forward extraction and a backward extraction. Proteins dissolve in the aqueous cores containing RMs during the process of forward extraction, and dissolved proteins are recovered from the RMs during the backward extraction. 

The extraction yield for the forward extraction of defatted wheat germ protein by RMs under ideal circumstances reached up to 37% [146,147]. By utilizing both RM and ultrasonic procedures, forward extraction can be enhanced by up to 57% [148]. When extracting proteins from grape seeds, recent research obtained a forward extraction rate of 82% [149]. In the traditional backward extraction procedure, the forward extraction solution is diluted by an equivalent amount of aqueous solution [150]. This approach comes with a number of drawbacks, including increased water and energy use, ineffective backward extraction, and surfactant and protein loss. Therefore, new backward extraction approaches are being thoroughly investigated by researchers [151].

#### 4.2.4. Subcritical Water Extraction

The technique of subcritical water (SW) extraction (SWE) involves using hot water between the level of the water’s boiling point (100 °C) and its critical point (374 °C) while applying considerable amounts of pressure in order to keep the water in liquid condition within the range of those temperatures. The characteristics of water are considerably altered by a rise in temperature. For instance, although its viscosity is low and its compressibility is decreasing, the most significant change is the temperature-dependent reduction in water’s dielectric constant. As a result, the water structure made of hydrogen bonds is compromised, allowing for the dissolution of both mildly non-polar and polar molecules. Additionally, SW creates a high-ion product, which has the ability to facilitate hydrolysis processes. As a result, in contrast to water that is at low temperatures and atmospheric pressures, SW parameters promote the breakdown of polysaccharides and the production of smaller solubilized protein fragments [152]. 

While highlighting production as the main obstacle to its commercial usage, it is a strategic option for the food business with considerable possibilities in the selective extraction of bioactive components and in green hydrolysis processes. SWE is a technique that is effective, affordable, quick, and ecologically friendly [153]. SW offers equivalent or greater yields in less time than traditional alkali or enzymatic hydrolysis. Researchers emphasize the need for creating ideal processing conditions as a workaround for using proteins produced from agro-food byproducts and biomass from algae, indicating that this might be advantageous in a stage-by-stage procedure for generating valuable components [154].

### 4.3. Novel Green Techniques of Extraction

The development of new extraction techniques has been prompted by the rising demand for plant proteins [133,134,135]. The selection of the extraction technique is a crucial element that affects the extraction quality and yield. Proteins can be extracted from plant matrices using both conventional and novel extraction techniques. Conventional methods are less common among researchers due to their lengthy duration and extensive use of toxic solvents. Additionally, concern for the environment has led to the development of novel green extraction techniques that produce a superior yield in a short amount of time with little solvent consumption. Some common novel green extraction techniques are discussed below [155,156,157].

#### 4.3.1. Microwave-Assisted Extraction

Microwaves, which typically process food, fall into the category of non-ionizing radiations and have a frequency that ranges from 300 MHz to 300 GHz [119]. In recent years, microwave-assisted extraction has grown in popularity as a method to extract active ingredients from food, especially when it comes to recycling waste products from agro-industrial processes [156]. 

Based on the electrophoretic transport of ions and electrons, microwave begins ionic conduction, creating an electric field [157]. Polar molecules are moved by dielectric heating in a time-dependent electric field, which forces the molecules to rotate into alignment with the electric field already present. These systems generate energy, which is then released as heat. Pressure from the loss of moisture inside the plant cell promotes expansion and eventual rupturing, which reveals the cell to the outside solvent and makes it easier for the solvent to penetrate [155]. This process produced 1.54 times as much protein as a chemical technique that used alkaline liquids. Due to the substantial amount of thermal energy produced by the microwave treatment, which degrades susceptible bioactive substances, several proteins are not extracted by this method. To effectively extract the plant proteins, another option is to use brief pulses of microwave energy or to optimize the microwave’s input settings.

#### 4.3.2. High Pressure-Assisted Extraction

High pressure-assisted extraction uses solvent extraction while operating at a high pressure of 100–1000 MPa [158]. As a result of being kept close to their supercritical regions under tremendous pressure, organic solvents stay in a liquid state [159]. These circumstances lead to a decrease in the solvent’s surface tension and density, which improves its absorption into the solid structure and increases the rate of mass transfer and mobility while shortening extraction times and solvent expenditure. 

The occurrence of high pressure causes air to leak in plant cell vacuoles, promoting the breakdown of proteins found in cell membranes and cell membrane breakdown, thereby enhancing the extraction permeability of the target compounds [160]. It was discovered that rice bran protein extraction was higher between 600 and 800 MPa and lower around 200 MPa. 

Despite the fact that high pressure cannot extract more protein on its own when combined with other effective methods like EAE, a protein-rich extraction rate of 66.3% could be achieved successfully [161]. It is becoming more and more popular as an alternative to traditional solvent-based extraction procedures because it is an environmentally friendly extraction technique. Applying HPAE in conjunction with enzymatic, ultrasonic, or microwave treatment can help recover plant proteins even better.

#### 4.3.3. Pulsed Electric Field-Assisted Extraction

The components of a pulsed electric field system include a high voltage origin, which is occasionally in the form of a switch, a capacitor bank, and an area with at least two electrodes [162]. A pulsed electric field is one that is created by applying quick electric pulses with high power (kV) at brief intervals (micro-milliseconds). An electric field potential is created when the dipolar cell membrane is exposed to an electric field, and this results in the separation of the electrical charge [163]. The creation of pores that allow for the transport of intracellular substances is known as electroporation, which occurs when the electric field strength exceeds the threshold range (0.8–1 V). Increased electric field strength and exposure time lead to more total-specific energy, which improves mass transfer and electroporation [164]. 

When rapeseed stalk debris was valorized for protein and polyphenol recovery employing a hydraulic press after having been pretreated with a pulsed electric field, the number of protein concentrations in the extracted juice was increased by double relative to the untreated biomass [129,165]. For the purpose of retrieving proteins in their original condition, it is necessary to optimize the input components of the pulsed electric field. As the protein grade is barely impacted during treatment and throughout the storage duration, it is an intriguing method in comparison with traditional heat procedures.

#### 4.3.4. Ultrasound-Assisted Extraction

Like all sound waves, ultrasounds propagate through the molecules of the material they are exposed to in a succession of compression and rarefaction waves. At high intensities, rarefaction cycles outweigh the molecule attraction forces of the medium, creating cavitation bubbles. When cavitation bubbles collapse, they create high-velocity jets that break cellular structures and make it easier for the solvent to penetrate [165]. 

Additionally, cavitation bubbles that burst produce heat energy, which improves mass transmission [166]. The cavitation, thermal, and mechanical effects of ultrasound during ultrasonic-assisted extraction lead to the breakage of cell walls, a decrease in particle size, and degradation of the plant matrix, as well as an increase in mass transfer [167]. Heat labile chemicals may be damaged by the heat produced during ultrasound-assisted extraction. Although the procedure uses less solvent, it is still problematic to employ harmful solvents like hexane, especially when extracting certain non-polar chemicals. Deep eutectic solvents, for example, are recognized as ‘green’ substitutes for harsh chemicals. Deep eutectic solvents have a high solvent capacity and are made from affordable, recyclable, and biodegradable materials.

Systematization of all previously mentioned extraction techniques can be summarized in the form presented in Table 4.

**Figure 2:**
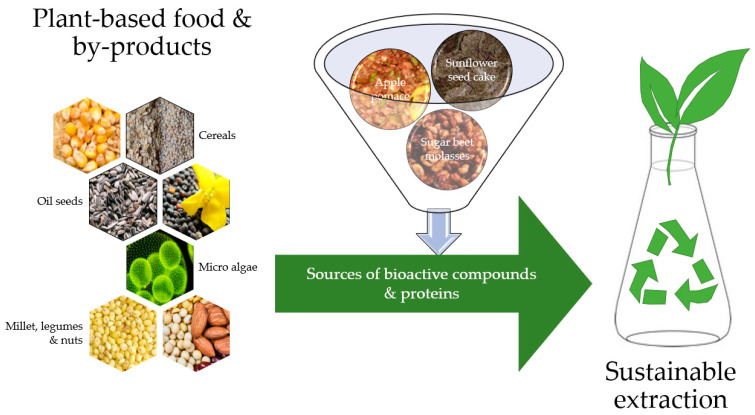
Plant-based food and their production byproducts as valuable and natural sources of bioactive compounds and proteins.

All of the previously mentioned needs to be in focus when the economic efficiency of the reprocessing of valuable byproducts of the plant-based food industry is evaluated. Green technologies will be in accordance with the strategies of “zero waste” in the food industry, with added value in the form of (i) environmentally friendlier products (ensuring a lower CO_2_ footprint and less pollution of water, air, and soil) as well as (ii) being more acceptable to the end user (consumers). By summarizing all that has been previously mentioned, the expected result that will be the output is shown in Figure 2.

## 5. Conclusions 

Food production, regardless of the origin (plant-based or not), is and will be a source of waste. But isolation of proteins and bioactive compounds from natural sources, such as plant-based food, is related to the ever-increasing demand for them in the pharmaceutical, chemical, and food sectors. When the above is extracted from a byproduct or waste, this factor gives added value to the new product for which it is planned to be used. Interest in natural products and products containing bioactive components from natural byproducts is growing proportionally to studies that confirm their effectiveness in the treatment and/or prevention of oxidative stress, inflammatory processes, and/or chronic diseases (e.g., metabolic syndrome, obesity), which has been proven in vitro or in animal models [55]. However, further studies are necessary, which would investigate the interaction of one or more bioactive components with medicinal ones through clinical trials.

The usefulness of conventional extraction methods, which we did not consider in this paper, should not be ignored. However, they are often time-consuming and sometimes environmentally unacceptable [171], and the aforementioned limitations led to the development of the unconventional extraction methods that we mentioned in the paper. However, like any measurement procedure, non-conventional methods also have disadvantages, of which high maintenance costs [172] and losses when extraction is carried out outside controlled laboratory conditions should be highlighted.

Unconventional methods have not yet sufficiently substantiated their justification through tests of usability, bioavailability, and bioavailability of such production practices.

Climate change and the pandemic of non-communicable diseases (diabetes, cancer, obesity) have affected the perception of sustainable products, considering that everything “green” and natural is also healthier [17,40,41,55,70,73], but at the same time there is an awareness that a number of scientific confirmations are needed. This points to the fact that the demand for food and food supplements derived from plants and algae will increase [173]. It is the projections of exceptional demand that will be the impetus for further research into sustainable and effective methods of screening, extraction, characterization, and processing of bioactive compounds and proteins, from plant sources, of good quality. Emphasis will be on research to improve extraction yield and selectivity with simulation modeling and optimization of interaction parameters and affinity of compounds and solvents, as well as work with biosensors, fluidic chips, etc. [174]. However, cost reduction and simpler scalability are expected through the optimization of physical parameters of non-conventional methods [171].

Nevertheless, this waste is a source of significant amounts of bioactive components and proteins that should be viewed through the lens of a circular bioeconomy, in which this is a potential that should be effectively monitored. However, for the future perspective, it is important to weigh the advantages and disadvantages, because the awareness of potential limitations (economic, ecological, ethical, etc.) will greatly affect the further development of the methods and the overall sustainability [175]. Using the example of bioactive components that have been found in grape pomace (and which are briefly discussed under Table 1), has clarified the importance of examination of the available and published data on the desired bioactive component. It is also important to carefully study the data on the diversity of the byproduct that remains in production, which comes from different sources, from different locations, etc. [43,56]. The study on the valorization of waste of plant origin [176] gained importance in the targeted sustainable development and minimization of the carbon footprint and greenhouse gas emissions, which is largely the result of the accumulation of waste. The content of bioactive compounds with functional properties, such as antioxidant and antibacterial properties, stimulated the development of the process of their “green” isolation. However, time-consuming and laboratory-intensive extraction protocols encourage the use of environmentally friendly solvents in response to consumer demand. Despite all of the above, this approach requires interdisciplinary research that includes food chemistry, food technology, biotechnology, molecular biology or toxicity, and nutrition. Namely, for the successful isolation of bioactive components and proteins from foods of plant origin, it will be imperative to improve in the following aspects: (i) “greener” extraction, (ii) open science that will provide information and data about the performed extractions and their efficiency and CO_2_ footprint; and (iii) economic aspects of the implementation of extractions. Hereby is also given an overview of the methods used in the sustainable “extraction” of bioactive components and proteins, originating from plant sources, where energy and ecological efficiency are not on opposite sides of the scale, but the known cons and pros should always be weighed in the final assessment.

## Figures and Tables

**Table 1:** Bioactive compounds from byproducts of plant-based food production (fruits and vegetables).

Food By-Product	Bioactive Compounds			References
	Class	Concentration (mg/kg)	Major Compounds	
	Vegetable By-Products			
Beetroot Pomace	Phenolic acids	1513	Ferulic acid, Caffeic acid, p-Hydroxybenzoic acid, Vanillic acid, Protocatechuic acid	[21]
	Flavonoids	386	Catechin, Epicatechin	
	Betalains	558.8	Betaxanthins, Betacyanins (isobetanin and betanin)	
Potato Pulp and Peel	Carbohydrates	/	Pectin	
	Glycoalkaloid	639–3580	α-Solanine, α-Chaconine	[22,23,24]
	Phenolic acids	1830–9130	Caffeic acid, Chlorogenic acid	
Broccoli leaves	Glucosinolates	1332–1594	Glucoiberin, Gluconasturtiin, Glucoraphanin, Glucobrassicin, Neoglucobrassin, 4-Methoxy-glucobrassicin	
Broccoli stalks and florets	Flavonoids	56.6	Quercetin, Kaempferol	[25,26]
	Phenolic acid	74.6–193.8	Neochlorogenic acid, Chlorogenic acid, Sinapic acid	
	Glucosinolates	1836.6–5775.6	Glucoiberiin, Glucoraphanin, Glucoalyssin, Neoglucobrassin, Glucobrassicin, Glucoerucin, Gluconapin	
Carrot Peel	Carotenoids	205.6	Lutein, α-Carotene, β-Carotene, Lycopene	
Cauliflower Stem and leaves	Phenolic acids	/	Sinapic acid, Ferulic acid	[27,28]
	Isothiocyanate	/	/	[29]
	Flavonoids		Quercetin, Kaempferol, Glycosides	[30]
**Fruit By-Products**				
Apple Pomace	Phenolic acids	523–1542	Caffeic acid, p-coumaric acid, Sinapic acid, Chlorogenic acid, Ferulic acid, p-coumaroylquinic acid	[31,32,33,34,35,36]
	Anthocyanins	50–130	Cyanidin-3-O-galactoside	
	Triterpenoids	/	Oleanolic acid, Ursolic acid	
	Carbohydrates	/	Pectin, Pectin oligosaccharides	
	Flavonoids	2153–3734	Isorhamentin, Quercetin, Glycoconjugates, Kaemferol, Rhamnetin, Epicatechin	
	Dihydrochalcones	688–2535	Phloretein, Phlorizin	
Grape pomace	Phenolic acids		Hydroxybenzoic acids (Gallic acid, Syringic acid); Hydroxycinnamic acids (Caffeic acid, p-Coumaric acid, Ferulic acid);	[37,38,39,40,41,42,43]
	Anthocyanins	13,169–78,537		
	Flavanols	1000–12,886	Procyanidin B1, (+)-Catechin, Procyanidin B2, (-)-Epicatechin, (-)-Gallocatechin, (-)-Gallocatechin gallate, (-)-Epicatechin gallate	
	Flavonols		Kaempferol-3-glucoside, Quercetin	
Plum Pomace	Flavonols	40.3	Quercetin, Kaempferol, Glycosides, Rutinoside	[44]
	Phenolic acid	95.7	Chlorogenic acid, Neochlorogenic acid	
	Anthocyanins	6.5	Cyanidin, Peonidin	
Mango Peel	Carotenoids	1900	β-cryptoxanthin, β-carotene, Lutein	
Mango Kernel Seed	Flavonoids	7200–13,000	Fisetin, Isoquercetin, Quercetin	[45,46,47]
	Xanthanoids	13,600	Mangiferin	
	Phenoic acids	/	Gallic acid	
	Catechins	/	Epicatecin, Epigallocatechin	
Berries Press Residue	Anthhocyanins	84,120 (blueberries) 27,890 (lingonberries) 284,950 (bilberries) 43,530 (cranberries)	Malvidin, Cyanidin, Petunidin, Delphinidin	
Banana Peel	Flavonols	1019.6	Rutin, Kaempferol, Laricitrin, Quercetin, Myricitin	[48,49]
	Catecholamines	4720	Dopamine	
	Phenolic acids	99.5	Ferulic acid, Sinapic acids, p-Coumaric acid, Caffeic acid	
	Catechins	/	Epicatechin, Catechin, Gallocatechin	
Citrus Peel and Pulp	Phenolic acids	560 (orange) 276 (lemon)	Caffeic acid, Hydroxybenzoic acid	[50,51]
	Flavanones	22298 (orange) 10646 (lemon)	Hesperidin, Eriocitrin, Narirutin	
	Flavones	55 (orange) 1659 (lemon)	Diosmetin glucoside, Apigenin glucoside	

**Table 2:** Bioactive compounds from byproducts of grain production.

Source	Bioactive Compounds	Concentration	Reference
Wheat Bran	Thiamin	0.65 mg/100 g	[58]
	Riboflavin	0.51 mg/100 g	
	Niacin	28 mg/100 g	
	Pantothenic acid	3.15 mg/100 g	
	Pyridoxine	1 mg/100 g	
	Folate	0.23 mg/100 g	
	Total Carotenoids	4.2 ug/g	[59]
	Lignan	4.75 mg/100 g	[58]
	Phytosterol	158 mg/100 g	
	Betaine	868 mg/100 g	
	Choline	172 mg/100 g	
	Total Flavonoids	3000–4300 ug/g	[60]
	Alkyresorcinol	489–1429 ug/g	[61]
	Phytosterols	4.73–2020 ug/g	[58]
	Ferulic acid	1376–1918 ug/g	[62]
	Total Phenolic content	4206.16 ug/g	[63]
Rice Husk	p-coumaric acid	265.4 mg/100 g	[64]
	Ferulic acid	33.64 mg/100 g	
	Total Flavonoids	3.08 mg CE/g	
	Total Phenolics	14.90 mg GAE/g	
	Caffeic acid	3.68 mg/100 g	
	p-hydroxybenzoic acid	12.55 mg/100 g	
Corn Bran	Total Phenolic content	1925 mg GAE/100 g	[59]
	Total Carotenoids	32.0 μg/g	
Corn Germ meal	Total Carotenoids	57.9 μg/g	

CE: Catechin equivalent; GAE: Gallic acid equivalent.

**Table 3:** Proximate protein composition of different plants.

Plant	Ref	Protein %	Glutelin %	Albumin %	Prolamin %	Globulin %
**Cereals**						
Maize	[87]	10.3	40	5	50	5
Rice	[88]	6–8	75–81	5–10	3–6	7–17
Wheat	[89]	8–13	42–62.5	15–20	28–42	15–20
Barley	[90]	10–12	25	2–3	30	2–3
Oats	[91]	16.9	35–40	20–25	10–15	20–25
**Oil seeds**						
Rapeseed	[92]	38	10	<1	20	70
Sunflower	[93]	20–40	17	38	5.5	39
			**Microalgae**			
*Cyanophyceae*	[94]	43–77 *	/	/	/	/
*Chlorophyceae*	[94]	11–55 *	/	/	/	/
**Millet, legumes and nuts proteins**						
Millet	[95]	9–13	30	15	30	8
Peas	[96]	25.6	19.1	13.8	3.08	57.2
Lupins	[97]	44	5.7–11.9	8.9–23.9	0.6–1.8	46.5
Soy	[98]	40	30.4	22.9	0.3	46.5
Lentils	[99]	26	2.1–3.5	56.3–64.0	1.4–2.0	26.5–29.5
Cashew	[100]	20–25	11.7	45.6	0.4	42.4
Pistachios	[101]	20	7.3	25	2	66
Almonds	[102]	22.7–29.9	<5	21	<5	74
Walnuts	[103]	18–24	72	7.5	4.7	15.7

* Protein content (% dry weight basis).

**Table 4:** Systematization of bioactive compounds and protein extraction techniques.

Extraction Method	Technique	Protein Yield/%	Ref
Dry Extraction	Air classification techniques	18	[165]
	Sieving	17	[166]
Wet Extraction	Aqueous two-phase system extraction	90	[167]
	Subcritical water extraction	80	[140]
	Reverse micelles extraction	37	[143]
	Enzyme-assisted extraction	74	[137]
Novel Green methods of Extraction	High pressure-assisted extraction	66.3	[134]
	Microwave-assisted extraction	24	[168]
	Ultrasound-assisted extraction	63	[169]
	Pulsed electric field-assisted extraction	25.4	[170]

## Data Availability

No new data were created or analyzed in this study. Data sharing is not applicable to this article.
